# Using Drift Diffusion Modeling to Understand Inattentive Behavior in Preterm and Term-Born Children

**DOI:** 10.1037/neu0000590

**Published:** 2019-10-03

**Authors:** Jenny Retzler, Chris Retzler, Madeleine Groom, Samantha Johnson, Lucy Cragg

**Affiliations:** 1 Department of Psychology, School of Human and Health Sciences, University of Huddersfield, and School of Psychology, University of Nottingham; 2 Department of Psychology, School of Human and Health Sciences, University of Huddersfield; 3 Division of Psychiatry & Applied Psychology, School of Medicine, University of Nottingham; 4 Department of Health Sciences, University of Leicester; 5 School of Psychology, University of Nottingham

**Keywords:** attention, very preterm, drift diffusion model, information processing

## Abstract

***Objective:*** Children born very preterm are at increased risk of inattention, but it remains unclear whether the underlying processes are the same as in their term-born peers. Drift diffusion modeling (DDM) may better characterize the cognitive processes underlying inattention than standard reaction time (RT) measures. This study used DDM to compare the processes related to inattentive behavior in preterm and term-born children. ***Method:*** Performance on a cued continuous performance task was compared between 33 children born very preterm (VP; ≤32 weeks’ gestation) and 32 term-born peers (≥37 weeks’ gestation), aged 8–11 years. Both groups included children with a wide spectrum of parent-rated inattention (above average attention to severe inattention). Performance was defined using standard measures (RT, RT variability and accuracy) and modeled using a DDM. A hierarchical regression assessed the extent to which standard or DDM measures explained variance in parent-rated inattention and whether these relationships differed between VP and term-born children. ***Results:*** There were no group differences in performance on standard or DDM measures of task performance. Parent-rated inattention correlated significantly with hit rate, RT variability, and drift rate (a DDM estimate of processing efficiency) in one or both groups. Regression analysis revealed that drift rate was the best predictor of parent-rated inattention. This relationship did not differ significantly between groups. ***Conclusions:*** Findings suggest that less efficient information processing is a common mechanism underlying inattention in both VP and term-born children. This study demonstrates the benefits of using DDM to better characterize atypical cognitive processing in clinical samples.

## General Scientific Summary

Less efficient information processing during a sustained attention task explained individual differences in inattentive behavior. This was true both in 8- to 11-year-olds born very preterm and their term-born peers. Drift diffusion modeling provides a way to help us better characterize the processes that underlie task performance. This is valuable for understanding processing differences that affect clinical groups.

One of the most common adverse outcomes from very preterm (VP; <32^+0^ weeks’ gestation) birth is attention-deficit/hyperactivity disorder (ADHD; Johnson & Marlow, 2011; Linnet et al., 2006), a condition characterized by developmentally inappropriate and functionally impairing levels of inattention and/or hyperactivity-impulsivity. Risk for ADHD in the VP population is two to three times greater than for children born at term (Johnson & Marlow, 2011). Notably, VP children are at significantly greater risk for inattentive symptoms than hyperactivity/impulsivity (Ask et al., 2018; Brogan et al., 2014), and mean symptom scores are significantly elevated even where children do not meet the threshold for diagnosis (Jaekel, Wolke, & Bartmann, 2013; Johnson et al., 2016; Johnson & Marlow, 2011). Given evidence showing that inattentive symptoms predict academic underachievement in both VP (Jaekel et al., 2013) and general population samples (Sayal, Washbrook, & Propper, 2015), and that inattention in the VP population persists into adulthood (Ask et al., 2018; Breeman, Jaekel, Baumann, Bartmann, & Wolke, 2016), inattention can be characterized as a core, lifelong impairment following VP birth. Increasing understanding of the processing deficits underlying inattentive symptoms may improve the ability to detect impairment and trial the suitability of interventions, to, ultimately, improve long-term outcomes for individuals born VP.

While ADHD in the general population is by and large recognized as the result of a gene-environment interaction (Faraone et al., 2005), it is thought that the increased risk for inattention in VP children arises as a result of aberrant neurodevelopment due to birth at very preterm gestations (Lindström, Lindblad, & Hjern, 2011). Specifically, there is evidence of atypical structural neural connectivity and white matter development, even in VP children who do not show major brain injury or impairment (Ment, Hirtz, & Hüppi, 2009), and associations have been observed between atypical white matter development and inattention in adolescents born VP (Skranes et al., 2007). With potentially differing initial causal factors, the mechanisms underlying inattentive symptoms in children born very preterm may be different from those in term-born children with ADHD. Exploring possible differences in these underlying mechanisms would inform theories that have postulated a “pure” form of inattention associated with VP birth (Hille et al., 2001). It would also be of clinical importance, with implications as to whether interventions currently available to treat ADHD are suitable in the VP population.

Slower and more variable response times (RTs) in speeded RT tasks have traditionally been considered as markers of poor attention, having been shown to differentiate between children with and without ADHD and to correlate with symptoms of inattention (e.g., Castellanos & Tannock, 2002; Hervey et al., 2006; Leth-Steensen, Elbaz, & Douglas, 2000; Uebel et al., 2010; Vaurio, Simmonds, & Mostofsky, 2009). Despite vastly increased measurement accuracy since the introduction of software with millisecond timing, standard RT measures remain limited in scope. Individual differences in RT may be attributable to differences in the speed of stimulus perception, decision-making, or the motor response. As such, the precise source of variation cannot be determined using standard RT metrics and the reliance on these measures may fail to identify specific cognitive processes underlying inattention. Moreover, if interactions between the different component processes have opposing effects on RTs (e.g., if inattentive children process information slowly, but make fast and impulsive decisions, while attentive children process information quickly but make slower decisions), the resulting RTs may mask the underlying processing differences.

**Figure 1 fig1:**
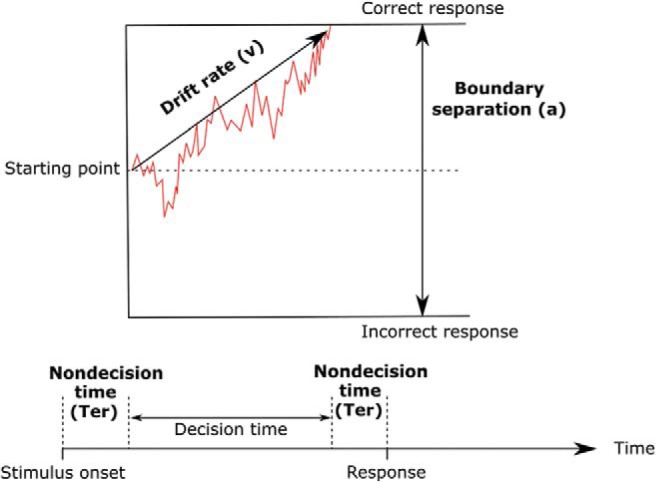
The EZ drift diffusion model of decision making (Wagenmakers, Van Der Maas, & Grasman, 2007) provides estimates of drift rate (*v*), boundary separation (*a*), and nondecision time (*Ter*).

The drift diffusion model (DDM; see Figure 1 for a graphical representation) uses intraindividual variability in RT and accuracy across trials to isolate underlying cognitive processes. The model assumes that the decision to make a response is a cumulative process in which noisy sensory information is gathered in favor of each response option, from a given starting point, until a decision threshold is reached (Ratcliff & McKoon, 2008). While the “full” DDM provides estimates of drift rate, boundary separation, nondecision time, starting point, and trial-to-trial variabilities in drift rates, nondecision time and starting point, the complexity of this model, both in terms of the structure of input data required and parameter fitting, reduces its applicability to many data sets, including that in the current study. A simplified version of the model (the EZ-DDM) has been developed that provides estimation of the most cognitively relevant of these parameters: drift rate, boundary separation, and nondecision time (Wagenmakers, van der Maas, & Grasman, 2007). Drift rate reflects the rate of information processing over time, referred to as information processing efficiency, as demonstrated by studies reporting lower drift rates in more difficult tasks compared with simpler tasks (Ratcliff & McKoon, 2008). Boundary separation reflects the speed–accuracy trade-off, or impulsivity, of the participant (Metin et al., 2013; Wiecki, Sofer, & Frank, 2013). When this threshold is low, the participant makes decisions after accumulating only a small amount of information, meaning decisions will be fast but less accurate, reflecting impulsivity, and when this threshold is high, the participant requires more information before making decisions, meaning they will be more accurate but slower, reflecting conservatism. Nondecision time provides a measure of the noncognitive elements of decision making such as encoding and response processes.[Fig-anchor fig1]

Use of DDM in clinical samples has demonstrated how this approach can alter the conclusions that would have been drawn using standard RT and accuracy measures alone (Pirrone, Dickinson, Gomez, Stafford, & Milne, 2017; Zhang, 2012). For example, Pirrone, Dickinson, Gomez, Stafford, and Milne (2017) used DDM analysis to show that despite slower responses by patients with autism spectrum disorder (ASD) on an orientation discrimination task, perceptual sensitivity was not impaired in this group, though behavioral data in isolation would have suggested it was.

Given the correspondence between the DDM measures and processes that have long been implicated in attentional processing, such as response speed, impulsivity, and response preparation, it is, perhaps, unsurprising that DDM has been used to assess impaired processing in ADHD. Analyses using case-control paradigms have consistently identified low drift rates as the primary explanation of group differences in task performance across a range of RT tasks, indicating that individuals with ADHD show less efficient processing. For example, Karalunas, Huang-Pollock, and Nigg (2012) found that group differences in RTs on a stop task were explained by drift rate, rather than boundary separation (speed–accuracy trade-offs) or nondecision processes such as response preparation. Similarly, Metin et al. (2013) found that those with ADHD showed lower drift rates and less nondecision time but no difference in boundary separation in both a simple choice RT task and a conflict control task, concluding that the RT differences in ADHD reflect inefficient information accumulation, rather than impulsive processing. Finally, Weigard and Huang-Pollock (2014) showed that in a contextual cueing task, individuals with ADHD, again, showed lower drift rates compared with controls, but also less flexibility in boundary separation.

To our knowledge, research into inattention using the DDM approach has been limited to case-control studies of ADHD to date, without thorough investigation of the extent to which the cognitive processes isolated by DDM can predict variation in symptom severity, nor investigation of the relationship of the parameters to particular symptom domains. Moreover, DDM has never before been used to investigate processing in individuals born VP, a population at risk for attentional deficits. Emerging evidence indicates that although many of the mechanisms underlying inattention are the same in term and VP samples, there may be specific deficits in processing speed that contribute to inattentive symptoms in children born VP (Mulder, Pitchford, & Marlow, 2011; Retzler et al., 2019). The evidence from these analyses is limited by the response and domain-specificity of the tasks, thus measures more sensitive to elucidating the underlying cognitive processes, such as DDM, may be useful to further understand how processing may relate to inattentive symptoms.

The current study investigates the value of DDM measures to further elucidate the cognitive mechanisms underlying inattention in VP and term-born children. A cued continuous performance task (CPT-AX), in which responses are required to infrequent cue-target sequences among distractor stimuli, was used to measure sustained attention. CPTs are known to be sensitive to the behavioral deficits observed in children with ADHD (Huang-Pollock, Karalunas, Tam, & Moore, 2012; Riccio & Reynolds, 2001), and studies have shown that task performance measures are best predicted by inattentive symptoms rather than hyperactive-impulsive symptoms (Chhabildas, Pennington, & Willcutt, 2001). To date, studies using CPTs to assess individuals born preterm have demonstrated poorer sustained attention in VP children relative to term-born controls (Mulder, Pitchford, Hagger, & Marlow, 2009), and shown that poorer task performance was associated with higher ADHD symptoms in VP adolescents (Rommel et al., 2017), but have not focused on associations between task performance and inattention specifically.

In order to facilitate the detection of correlates of inattention within both groups of children, and to directly compare these correlates between groups, term-born children were not recruited using a typical case-control approach. Instead, both groups were recruited to include children with a wide range of inattentive symptoms, as rated by their parents, ranging from above average attention to severe inattention (see online supplementary material and Retzler et al., 2019 for details). Accordingly, a dimensional measure of inattentive symptoms suitable for use in nonclinical samples and sensitive to the full range of attention scores was selected to capture the full range of these traits in both groups (the strengths and weaknesses of ADHD and normal-behavior [SWAN]; Polderman et al., 2007; Swanson et al., 2012).

Given that inattention is one of the core deficits in ADHD, it was predicted that, in line with previous studies in ADHD groups, drift rate would explain significant variance in inattention. As the first study to isolate cognitive components using DDM within a VP sample, our second hypothesis was two-tailed; we predicted that either inattention would be explained by the same DDM parameters in both groups, or that groups would differ on one or more parameters.

## Method

### Ethical Standards

Ethical approval was granted by a United Kingdom NHS Research Ethics Committee (Coventry and Warwickshire; Ref: 13/WM/0203) and informed parental consent was obtained for all children.

### Participants

Sample recruitment is described in detail in Retzler et al. (2019) and a full description of all children tested is presented in the online supplementary material. In brief, following identification from hospital records and tracing of all babies born VP (≤32 weeks’ gestation) and admitted for neonatal intensive care in Nottingham University Hospitals NHS Trust, 65 children were recruited (16% of eligible births) to the study. As a comparison group, 48 term-born children (≥37 weeks’ gestation) were then recruited from the same geographical area, using advertisements distributed via local schools and in the community, as well as the University of Nottingham volunteer database. This was a two-stage process that screened for inattentive symptoms using the parent-rated SWAN scale (Stage 1), before inviting families to participate in the full study (Stage 2). This process ensured that the seven points on the SWAN scoring scale were represented in the term-born children, reflecting a range of attentional abilities (far below average, below average, slightly below average, average, slightly above average, above average, and far above average).

The subsample for the current analysis comprised all children with available task data suitable for the DDM analysis (see online supplementary material for full explanation of why data for some children were unavailable). Ten children in each group achieved a 100% hit rate, which prevents calculation of DDM parameters and rendered their data unsuitable for the analysis. This resulted in a subsample of 32 term-born children and 33 children born very preterm aged 8–11 years. Comparisons of the children included in the DDM analysis with those not included, revealed no differences in gestational age at birth, sex, ethnicity, socioeconomic status (as measured using the English Indices of Multiple Deprivation; McLennan et al., 2011), IQ, or scores of parent-rated inattention and hyperactivity (*p* > .1 in all cases; see online supplementary material).

Moreover, the VP children in the subsample for this analysis did not differ significantly from the wider eligible VP children (*n* = 374; excluded either due to nonrecruitment to the study, or due to unsuitable data for the DDM) with respect to gestational age (*p* = .34), birth weight (*p* = .46), sex (*p* = .41), or socioeconomic status, (*p* = .19).

### Experimental Task

Children were asked to complete a CPT-AX programmed using PsychoPy software (Peirce, 2007) while electroencephalography (EEG) measurements were recorded as the last part of a test battery (EEG data not reported here). Children were seated at a desk in a quiet, unlit room facing a computer screen while wearing the EEG recording cap. An experimenter remained with them in the testing room at all times.

**Figure 2 fig2:**
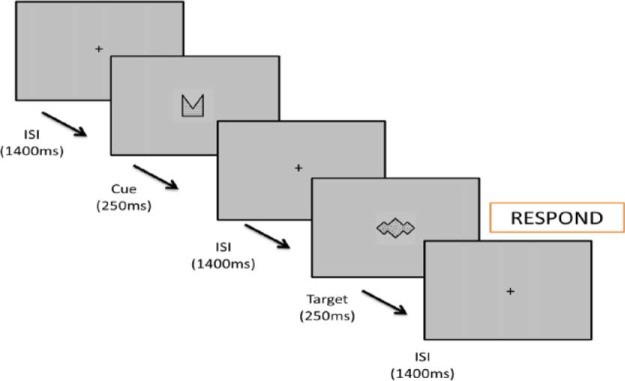
Schematic showing a cue-target sequence for the CPT-AX task.

At the start of the task written instructions appeared on the screen to familiarize the children with the stimuli that represented cues and targets. The stimuli consisted of black abstract shapes (chosen so that they did not have a verbal label) filled with different patterns presented on a gray background (see Figure 2). One stimulus was designated as the target stimulus (in CPT-AX nomenclature, this represents the X stimulus) and one stimulus as the cue stimulus (in CPT-AX nomenclature, this represents the A stimulus). The same shapes were designated as cue and target for all children. The instructions were read out by the experimenter who told each child that they were required to respond as quickly as possible when they saw a cue-target sequence. They were informed that the cue shapes and target shapes might also appear in isolation and it was reiterated that it was only when they saw a cue-target sequence in the specified order that they needed to respond.[Fig-anchor fig2]

A continuous stream of stimuli was presented in the center of the screen. Each stimulus was presented for 250 ms separated by an interstimulus interval of 1,400 ms, during which a central fixation cross was displayed (see Figure 2). A cue-target “go” (A-X) trial was defined as a trial-pair where the stimuli designated as the cue and target were presented consecutively. Each time the child saw the target stimulus sequentially following the cue stimulus, they were required to respond as quickly as possible pressing the left-most button on a Cedrus RB-730 button box with their right hand. Children were instructed to keep their finger over the response button so that they could respond as quickly as they could. No response was required to other trial types, including those where the cue and target were presented in isolation from one another.

The task consisted of four blocks of 100 trials, with the cue stimulus, target stimulus and 11 different distractor stimuli presented. Trials were presented in a pseudorandomized order, with different orders for each block, but identical orders across participants. “Go” (A-X) cue-target sequences were presented 10 times within each block, as were cue-without-target “no-go” trials (A-not-X), and uncued-target “no-go” (X-not-A) trials. On “go” trials, participants were required to respond within 1,650 ms of stimulus onset (prior to the presentation of the subsequent stimulus) to be considered “correct.”

### Standard Task Performance Measures

#### Hit rate

The total number of correct hits (responses made within 200–1,650 ms from the onset of a cued target) was summed as a measure of accuracy. This was reported as a percentage of correct hits out of the maximum score of 40. Higher scores represent more accurate performance, and thus better attention.

#### Commission errors

The total number of responses made on “no-go” trials (any trial other than a cued target) was summed as a measure of commission errors. This was reported as a percentage of erroneous responses out of the 360 “no-go” trials (error rates were too low to permit differentiation between type of “no-go” trial). Higher scores represent less accurate performance and therefore greater impulsivity.

#### Response time

The mean response time on correct hit (A-X) trials was calculated as a measure of response speed. Higher values represent slower response speed.

#### Response time variability

Finally, the standard deviation of response time on correct hit trials was calculated as a measure of response speed variability. Higher values represent greater variability in response speed.

### Participant Characteristics and Clinical Symptoms

An age-standardized estimate of full scale IQ (FSIQ-2) was calculated from the Wechsler Abbreviated Scale for Intelligence (Wechsler, 1999) using the vocabulary and matrices subtests. Inattentive and hyperactive-impulsive behaviors were measured using the raw scores on the SWAN rating scale (Swanson et al., 2012) a parent-report measure of a child’s ADHD symptoms. This scale has been considered appropriate for use in community populations (Swanson et al., 2012) as it allows measurement of variation in above average attention in addition to below average attention (more severe inattention; Arnett et al., 2013). To characterize inattention and hyperactivity in more clinical terms, measures of symptoms and risk of ADHD were assessed using the Conners 3-P (Conners, 2008), with higher scores indicating greater symptoms. Children with scores above the predefined clinical cut-off were classified as “at risk” of diagnosis.

### Diffusion Model Fitting

In light of the infrequent target stimuli in this task and consequent low trial numbers (*n* = 40), as well as the fact that error rates on “go” trials were low, the EZ-DDM (Wagenmakers et al., 2007) was used. The EZ-DDM is a simplified version of the full drift diffusion model and is ideal for use both with small numbers of trials, and with low numbers of error rates (Wagenmakers, van der Maas, Dolan, & Grasman, 2008).

The EZ-DDM (Wagenmakers et al., 2007) was fitted to the “go” RT and accuracy data from our task using custom R scripts (Wagenmakers provides customizable scripts for R here: https://www.ejwagenmakers.com/2007/EZ.R). The EZ-DDM transforms a participant’s overall accuracy, mean RT, and variance in RT using three equations derived from the full DDM (see Wagenmakers et al., 2007 for a more detailed description of the procedure). This simplification allows calculation of the most cognitively salient DDM measures without complex parameter fitting procedures often requiring large numbers of trials. This comes at the cost of a more detailed account of behavior provided by the full DDM which includes measures of cross-trial variability and starting point. For the purposes of EZ-DDM, such parameters are kept constant.

The EZ-DDM provides estimates of drift rate, boundary separation, and nondecision time parameters for each participant. For drift rate, a higher value indicates that more information is processed per unit of time (higher drift rate indicates more efficient information processing); for boundary separation, a higher value indicates greater separation between decision boundaries and thus a more conservative approach to decision making based on greater evidence accumulation, conversely a lower value suggests an impulsive decision. Finally, for nondecision time, a higher value indicates greater time spent encoding the stimuli and preparing and executing responses.

Prior to fitting, response times below 200 ms were rejected as these are likely to reflect anticipatory responses from participants prior to cognitive processing of the current stimuli (Ratcliff & McKoon, 2008).

To test the goodness of fit of the DDM model, 1,000 trials for each group were simulated using the “multisimul” function in the DMAT toolbox for MATLAB. The simulated data were created using model parameters (drift rate, boundary separate, nondecision time) averaged across individuals within each group to create mean parameters. Using these parameters, DMAT was used to create two simulated supersubjects. The mean RTs and accuracy from the observed data were then compared with those of our simulated data.

### Statistical Analysis

As children in both groups presented with a range of levels of parent-rated inattention, group differences in cognitive performance were not expected, but were analyzed to provide context. Group differences in CPT-AX performance were examined using a MANCOVA with group (term-born or VP) as the between-subjects factor and age entered as a covariate as this differed between groups.

To assess the pattern of association between parent-rated inattention and task performance and DDM measures, first partial correlations controlling for age were performed, both across groups and split by group. Next, in order to assess the independent contribution of these variables for explaining the variance in parent-rated inattention, any task performance or DDM measure that showed a significant correlation with parent-rated inattention in either term-born or VP children was entered into a hierarchical multiple regression, with parent-rated inattention as the dependent variable. Group and age were entered into the first step, and hit rate, RT variability, and drift rate were entered in the second step. Due to high intercorrelations between task performance and DDM measures (see online supplementary material), when these were entered in the second step, a data-driven stepwise-entry selection technique was used so that only those variables that added significant variance above and beyond that accounted for in the preceding steps were entered. This approach has been used previously (Aarnoudse-Moens, Weisglas-Kuperus, Duivenvoorden, van Goudoever, & Oosterlaan, 2013) to better separate out effects among variables that are interrelated. In order to investigate any group-specific effects, group interaction terms were included as predictor variables in a regression analysis. However, to assess whether the interaction terms explained additional variance while accounting for the loss of any task performance or DDM measures in the second step of the analysis due to the use of stepwise-entry variable selection, a separate regression analysis was conducted using forced entry technique at all steps, in which group interaction terms were added in a final step.

## Results

### Sample Characteristics

A full comparison of sample characteristics between VP and term-born children included in the DDM analysis are provided in the online supplementary material (Table SA1), with key features summarized in Table 1 below. Compared with term-born children, VP children were significantly older (*p* = .031) and had significantly lower IQ (VP: *M* = 99.2 points, *SD* = 14.0 points; term: *M* = 111.6, *SD* = 9.7 points; *p* < .001) but were well-matched on most other variables (see Table SA1). In spite of both samples including children with a large range of scores on the SWAN inattention scale, VP children had significantly more severe parent-rated inattention, than those in the term-born sample (VP: *M* = −0.70, *SD* = 9.89; term: *M* = −6.58, *SD* = 12.23; *p* = .038). That noted, while there were high correlations between Conners 3-P ratings of inattention and hyperactivity and SWAN ratings of inattention and hyperactivity (inattention *r* = .78, *p* < .001; hyperactivity *r* = .71, *p* < .001), there were no differences between groups in the number of children scoring “at risk” according to clinical cut-offs for *DSM* ADHD diagnoses on the Conners 3-P.[Table-anchor tbl1]

### Model fit

**Figure 3 fig3:**
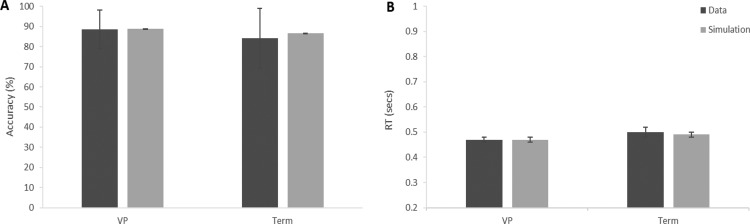
Comparison of simulated (light gray) and observed data. A shows mean percent accuracy for the VP and term born children. B shows the mean RT for correct responses for each group. Error bars are the standard error of the mean.

Our simulation showed that the model fit the data well with little difference between the simulated and observed data for either group (Figure 3; RT quantiles are presented in Figure SA1 in the online supplementary material for further detail).[Fig-anchor fig3]

### Between-Group Differences in Task Performance

Using Pillai’s Trace, multivariate tests showed that there was no significant effect of age (*V* = 0.121), *F*(7, 56) = 1.104, *p* = .373, or group (*V* = 0.102), *F*(7, 56) = 0.913, *p* = .504, on CPT-AX performance using either standard or DDM measures, therefore no univariate tests were conducted (see Table 2).[Table-anchor tbl2]

### Relationships With Parent-Rated Inattention

Partial correlations between task performance and DDM parameters and parent-rated inattention showed that across groups higher levels of inattention were associated with poorer hit rate, increased RT variability and a lower drift rate (see Table 3). The same pattern of findings was observed in both groups, although the correlation between RT variability and inattention did not reach significance in the VP group. Fisher’s comparison confirmed that there were no significant differences in the magnitude of correlations between groups for hit rate (*z* = 0.22, *p* = .83), RT variability (*z* = −1.35, *p* = .18) or drift rate (*z* = 0.32, *p* = .75). Full correlations between all task performance and DDM parameters and inattention are presented in the online supplementary material (see Table SA3).[Table-anchor tbl3]

The results of the hierarchical stepwise regression are reported in Table 4. Age and group alone did not explain significant variance in parent-rated inattention, *F*(2, 61) = 2.522, *p* = .089. The model was significantly improved with the addition of task-performance measures (Δ*R*^2^ = 0.153, *p* = .001), though only drift rate contributed enough unique variance to be entered into Model 2, which explained 22.9% of the variance in inattention (Model 2; *F*(3, 60) = 5.956, *p* = .001). Notably, with the inclusion of task-performance measures, group also explained significant unique variance in this model.[Table-anchor tbl4]

**Figure 4 fig4:**
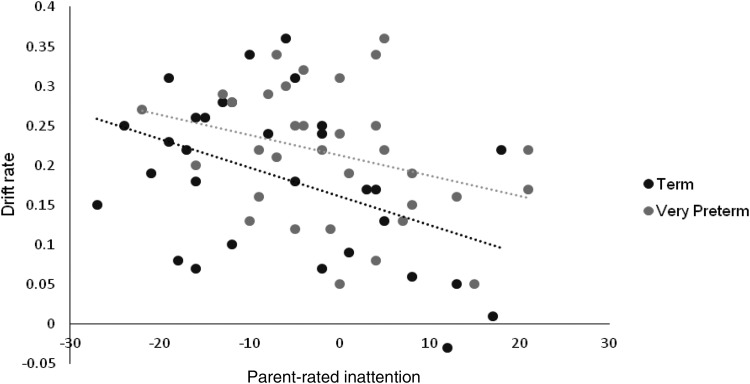
Scatter plot showing the association between parent-rated inattention and the drift rate parameter of the drift diffusion model for term and very preterm groups. Inattention scores of zero reflect an average level of attentive behavior, positive scores reflect a poorer than average level of inattentive behavior, and negative scores reflect an above average level of attentive behavior. Higher drift rate scores reflect processing of a greater amount of information per unit of time in favor of the “go” response (i.e., more efficient processing).

Finally, group-specific effects were assessed by conducting a separate regression analysis which used a forced entry variable selection technique throughout and added interaction terms in a third step. Addition of group-interaction terms did not explain significantly more variance than was explained by task performance and DDM measures alone, (Δ*R*^2^ = 0.039, *p* = .374), and none of the group interaction terms explained unique variance, thus no further analyses of group-specific effects were conducted and Model 2 (see Table 4) was accepted as the final model (see Figure 4).[Fig-anchor fig4]

## Discussion

### Findings and Implications

The aim of the current study was to explore the value of DDM measures for understanding the cognitive mechanisms underlying inattention in VP and term-born children. Unlike in a typical case-control study, we did not necessarily expect task performance differences between groups due to the range of attentional abilities of the children included in both groups, and the fact that they were well-matched for number of children with clinically relevant ADHD symptoms. Indeed, the groups did not differ on standard task performance metrics, nor on DDM measures. Across groups, more severe parent-rated inattention was associated with a lower hit rate, more variability in response time, and, as hypothesized, a lower drift rate (less efficient information processing). No significant differences between the two groups were observed in the magnitude or direction of correlations between task-performance or DDM measures and inattention. Across the sample, the best predictor of parent-rated inattention was drift rate, indicating that the DDM estimate of processing inefficiency best characterized the features of CPT task performance that related to inattentive behavior in our sample. Interestingly, parent-rated inattention was not predicted by boundary separation or nondecision time in either group, in line with converging findings from previous research that emphasizes the role of inefficient processing in ADHD (Karalunas et al., 2012; Metin et al., 2013; Weigard & Huang-Pollock, 2014).

In DDM, the extent to which the different parameters relate to standard task performance metrics depends on a combination of task demands and features of samples, so in some tasks, differences in RT between conditions or samples may be explained by differences in boundary separation, while in others, it may result from changes in nondecision processes. In our data, hit rate and drift rate were very highly correlated (see Tables SA2 and SA3 in the online supplementary material), which supports previous evidence showing that drift rates are often substantial drivers of accuracy (Ratcliff & McKoon, 2008). In fact, running the same regression analysis without the drift rate parameter resulted in a model where hit rate was the only task performance measure retained (see Table SA4 in the online supplementary material), and a model that explained a similar amount of variance to Model 2 above (which does include drift rate). The benefit, therefore, of using the DDM parameters is an ability to better understand the cognitive processes behind the task performance, as opposed to greater explanatory power. A logical interpretation of hit rate predicting inattention is that children who have more severe inattentive behavior are less accurate in the CPT-AX, missing more of the infrequent target stimuli. We do not know what processing deficits lie behind this inaccuracy. It could be due to response preparation being so slow that targets are missed (this would be captured by the parameter of nondecision time), or it could be due to poorly calibrated speed–accuracy trade-off (which would be captured by the parameter of boundary separation). In this instance, the parameter of drift rate emerges as key. Accordingly, interpretation of the relationship between drift rate and inattention provides us with a clearer narrative. Children with more severe inattention are less efficient at information processing, which is likely one of the factors that contributes to a reduced hit rate.

Another advantage of exploring DDM measures when characterizing behavioral deficits is that there is accumulating evidence of neurological areas and processes associated with specific DDM measures, which can help us to understand more about the causal pathway from brain to behavior. The drift rate parameter has been causally linked to the dorsolateral prefrontal cortex (DLPFC), an area thought to integrate sensory evidence (Philiastides, Auksztulewicz, Heekeren, & Blankenburg, 2011) and implicated previously in ADHD, both in terms of reduced activation and delayed maturation of cortical thickness (Bush, 2010). Given this link, Karalunas et al. (2012) speculate that low drift rates in ADHD samples may be due to deficits in DLPFC function, resulting in difficulty in deciding between two response options. Such use of modeling techniques thus promises to aid in the identification of risk markers and intervention targets for inattention and other disorders in which inattention is a key component.

These results have implications for theories of ADHD. For some time, the focus has been on executive top-down explanations of the disorder but these findings add to a growing literature suggesting that ADHD may, at least in part, reflect deficits in the processing or accumulation of information which underlie the executive deficits (Rommelse et al., 2007). As acknowledged by Metin et al. (2013), these findings do not exclude the possibility that executive deficits are important, rather they indicate that executive dysfunction cannot be considered the sole cognitive factor in ADHD symptoms. Future research into inattentive behavior, both in term and preterm populations, should focus on using tasks with greater numbers of trials that would allow the fitting of more sophisticated DDM models with more parameters, to understand in more depth those cognitive processes underlying inattentive behavior.

The current analyses can be interpreted to indicate overlapping etiology of inattention in our two groups (though larger sample sizes would be required to provide sufficient power to rule out smaller effect size differences and provide certainty of equivalence^1^) which may have positive implications for the suitability of assessment and treatment options used in the general population for children born VP. Research assessing intervention effectiveness within the VP population would be required to explore this in greater detail, not only in light of the narrow scope of the current analysis, but also in recognition of the possibility that neurocognitive differences unrelated to inattentive behavior may still affect treatment response.

The analysis did not provide further evidence of differences between the cognitive processes relating to inattention in term and VP children, suggesting that the CPT taps into different processes to those implemented in Mulder, Pitchford, and Marlow (2011) and Retzler et al. (2019). To some extent, these contrasting findings demonstrate the precise difficulty that computational analysis of behavioral data aims to resolve. Interpretation of task performance in cognitive neuroscience is limited to differing degrees by the impurity of the measures employed, and is made more difficult still by the nonspecificity of terminology adopted. Both Retzler et al. (2019) and Mulder et al. (2011) used very different tasks that were both purported to measure some element of processing speed, finding that only VP children showed an association between these measures and inattention. It may, perhaps, seem contradictory for no similar finding to be observed in the current analysis with regard to drift rate, which is often referred to as processing efficiency, particularly given that it involves a subsample of those in Retzler et al. (2019). Further research that uses designs, techniques, and analytical approaches more sensitive to these underpinning cognitive processes is required to address this complexity, and to understand more fully any processing deficits that are specific to children born VP.

### Limitations

This study is limited by the use of the EZ-DDM in preference to more complex DDMs that are able to estimate a wider range of parameters. This decision was taken due to the limited number of “go” trials in the task, which resulted from a combination of the CPT-AX design (10% frequency of “go” trials), and consideration of the total number of trials it would be ethical and pragmatic to include for one task within an extended test battery for a sample with attention difficulties. The EZ-DDM is better suited to small numbers of trials (Wagenmakers et al., 2007). Indeed, previous research has demonstrated good fits with very few trials (Lerche, Voss, & Nagler, 2017; Pirrone et al., 2017) and our simulated data suggested a good fit to our empirical data. However, the EZ-DDM did not allow us to look at some of the other components of the DDM such as starting point of evidence accumulation or cross trial variability of model parameters. While the measures provided by the EZ-DDM are those considered the most psychologically relevant (Wagenmakers et al., 2007), and also include those identified by previous literature to underlie inattention, the inflexibility of EZ-DDM may have limited the accuracy of the estimation of our parameters. Starting point, in particular, may have been biased toward the more frequent “no-go” response in this task, and ideally, future studies should aim to collect data from enough trials to fit a full DDM. Inclusion of the other parameters of the full DDM may not only improve the accuracy of parameter estimation all-round, but may also avoid misinterpretation of data as demonstrated in Ratcliff, Huang-Pollock, and McKoon (2018). Incidentally, the EZ-DDM was created by Wagenmakers, van der Maas, and Grasman (2007) to provide an easily applied model that could be used by those without in-depth knowledge of modeling. The current findings, along with previous research in ASD (Pirrone et al., 2017), Parkinson’s disease (Zhang, 2012), and ADHD (Karalunas et al., 2012; Metin et al., 2013; Weigard & Huang-Pollock, 2014), highlight the worth of such an approach alongside standard analyses.

While the EZ-DDM was the best fit for our data as it can be used even with low trial numbers and error rates, it does still require *some* errors. Although Wagenmakers et al. (2007) proposed a correction method for cases of 100% accuracy, this has been critiqued by Ratcliff (2008) for unreliable parameter recovery, particularly with small trial numbers. As such, we opted to exclude the 10 children from each group who had achieved 100% hit rates, and had, unsurprisingly, also performed significantly better on other measures of task performance. Future research may benefit from manipulating task difficulty, for example, by making target and distractor stimuli visually similar in order to increase error rates, so that data for the full sample can be analyzed.

In contrast to the full study sample, SWAN scores in inattention differed between term and VP children in this subsample (see the online supplementary material for details), and group was a significant unique predictor of inattention when drift rate was modeled. However, given the dimensional approach used in the study design, matched samples per se were not necessary for achieving the study aims. As intended from the recruitment process, both groups in the subsample included children from the full spectrum of SWAN scores, facilitating detection of relationships between cognitive processes and inattentive behavior. Although there were group differences in SWAN inattention overall, there were no differences in the number of children scoring above clinical cut-offs on the Conners 3-P, suggesting that the number of children with clinically relevant symptoms was well-matched. This may explain why although drift rate was associated with SWAN inattention, which differed between groups, drift rate itself did not differ between groups.

Finally, in the current study the primary factor underlying inattention was the same for both groups. However, these results only provide a snapshot of the children’s development (between 8- and 11-years-old), thus it remains unclear whether the developmental trajectories underlying inattention are the same for those born VP and at term, or whether similarities observed here have been reached via distinct pathways. In order to fully understand the similarities and differences in the causal pathways to inattention and ADHD in preterm and term-born children more comprehensive studies are required. While these results provide an encouraging avenue for future research, it is important to note that the amount of variance in inattention explained by our model remained modest at 22.9%, suggesting that these differences in cognition were not the only factors involved in the etiology of inattention in this sample.

## Conclusions

In summary, the cognitive mechanisms underlying inattention in term-born and VP children seem to be at least partly overlapping. High levels of inattention were predicted by lower drift rate, indicating that inattentive behavior is associated with less efficient processing of information. Use of DDM parameters provided a better characterization of the individual differences in cognition that related to inattentive behavior in term and VP children.

## Supplementary Material

10.1037/neu0000590.supp

## Figures and Tables

**Table 1 tbl1:** Characteristics of Term-Born and Very Preterm Children

Participant demographics	Very preterm	Term	*p*
Gestational age at birth (weeks)^a^; mean (*SD*)	29.6 (1.9)	40.0 (1.2)	—
Birth weight (kg); mean (*SD*)	1.40 (.47)	—	—
Age at assessment (years); mean (*SD*)	9.6 (1.0)	9.1 (1.1)	.031*
Sex; % female	45.5	40.6	.694 *n.s.*
Conner’s 3 scores above clinical cut offs for *DSM* ADHD/I; *n*(%)	12 (36.4%)	7 (21.9%)	.199 *n.s.*
Conner’s 3 scores above clinical cut offs for *DSM* ADHD/C; *n*(%)	10 (30.3%)	10 (31.3%)	.934 *n.s.*
*Note*. *SD* = standard deviation.			
^a^ Five children (15.2%) in the VP sample were born at gestations of fewer than 28 weeks, meeting criteria for extremely preterm birth. *n.s.* = not significant.			
* *p* < .05.			

**Table 2 tbl2:** Age Adjusted Marginal Means and Standard Errors (SE) for Performance Measures of Term-Born and Very Preterm Children

Measure	Very preterm		Term	
	Mean	*SE*	Mean	*SE*
Commission errors (%)	2.4	.5	2.5	.5
Hit rate^a^ (%)	88.3	2.2	84.6	2.3
RT^a^ (ms)	478	15	492	15
RT variability (ms)	168	11	169	11
Drift rate	.211	.016	.191	.016
Boundary separation	.112	.004	.112	.004
Nondecision time	.253	.013	.267	.013
*Note*. *SE* = standard error of the mean; RT = response time; ms = milliseconds.				
^a^ See Figure SA2 in online supplementary material for a graphical representation of RT and accuracy quantiles by group.				

**Table 3 tbl3:** Partial Correlations Between Parent-Rated Inattention and Task-Performance

	Inattention		
	Collapsed across groups (*N* = 64)^a^	Very preterm (*N* = 33)	Term (*N* = 31)^a^
Commission errors	.241	.167	.319
Hit rate	−.350**	−.369*	−.418*
Response time	.152	.126	.210
RT variability	.318*	.163	.475**
Drift rate	−.369**	−.364*	−.435*
Boundary separation	.021	−.128	.136
Nondecision time	−.107	.004	−.159
*Note*. All correlations have been controlled for the effect of age.			
^a^ SWAN Inattention was not measured for one term-born participant.			
* *p* < .05. ** *p* < .01.			

**Table 4 tbl4:** Regression Model for Cognitive Predictors of Parent-Rated Inattention

Predictor	Inattention	
	Model 1	Model 2
	*R*^2^ = .076	*R*^2^ = .229*** Δ*R*^2^ = .191***
	β	β
Group	.230	.277*
Age	.099	.159
Drift rate		−.401***
Hit rate		—
RT variability		—
*Note*. — = Did not meet criteria for stepwise entry model selection.		
* *p <* .05. *** *p* < .001.		

## Footnotes

1 To further assess equivalence between groups we conducted some Bayesian analyses in addition to our null-hypothesis significance testing (NHST) inferential statistics. First we performed a Bayesian regression to calculate Bayes factors in favor of the alternative hypothesis for the prediction of inattention in a model with group, age, and drift rate as predictors. In line with our NHST analysis, we found strong evidence in favor of accepting the alternative hypothesis (BF_10_ = 25.95). To examine group-specific effects of drift rate on inattention, we performed a second analyses in which group, age, and drift rate were added to the null model (akin to including these variables in first steps of a hierarchical regression analysis), before calculating the Bayes factor for the inclusion of the group*drift rate interaction term. This provided some evidence in support of the null hypothesis (BF_01_ = 2.71), indicating that it is unlikely that the relationship between drift rate and inattention is group-specific.
