# Supplementary material for: Using Drift Diffusion Modeling to Understand Inattentive Behavior in Preterm and Term-Born Children
Source: Neuropsychology. 2019 Oct 3;34(1):77–87. doi: 10.1037/neu0000590 (PMC6939604; doi:10.1037/neu0000590)
Supplement: Supplementary file 1 [file Supplementary_neu0000590.docx]

**Supplementary material**

Details of study recruitment are described in full in Retzler et al. (2019), but see Figure SA1 for a brief infographic explaining the participant flow from initial recruitment to the inclusion of participants in the drift diffusion model (DDM) analysis sub-sample. The full study sample comprised 65 children born very preterm (VP) and 48 children born at term. Of these 22 VP and 6 term-born children did not complete the CPT-AX task on which this analysis is performed (due to time constraints (9 VP and 3 term), technical issues (4 VP) or intolerance to the EEG fitting procedure (9 VP and 3 term)). A further 10 children in each group achieved a 100% hit rate, preventing calculation of DDM parameters in these children. The resulting final sub-sample for this analysis comprised 33 VP and 32 term-born children.

**Figure SA1 Participant flow from recruitment to DDM analysis**

Tracing of 407 eligible very preterm children

296 very preterm children invited to take part in the study

Consent gained for 94 very preterm children

Deceased 1

Moved away 8

Not contacted 102

No consent 202

given

65 very preterm children tested

Withdrawn 8

Unable to 21

schedule

43 very preterm children completed CPT-AX

DDM parameters were calculated for 33 very preterm children

Time constraints 9

Technical issues 4

Intolerant to EEG 9

100% hit rate 10

48 term-born children tested

Study advertised to term-born children

Parents of 124 children completed the screening survey

Not contacted 28

Withdrawn 5

No response/ 43

unable to

schedule

42 term-born children completed CPT-AX

DDM parameters were calculated for 32 term-born children

Time constraints 3

Intolerant to EEG 3

100% hit rate 10

Table SA1 reports sample characteristics for children in the full sample and those included in the DDM analysis sub-sample, and the differences between term-born and VP children in each. Differences between children from the full sample who were included vs. not included in the DDM analysis were assessed using *t*-tests for continuous variables, and chi-squared analysis for categorical variables. Children included in the DDM analysis did not differ from those not included in this analysis on gestational age at birth, sex, ethnicity, socio-economic status, IQ, Conner’s 3 and SWAN scores of inattention and hyperactivity (*p*>0.1 in all cases). However, there was a marginal effect of chronological age, whereby those included in the analysis were marginally younger (M=9.3 years, SD=1.0 years) than those excluded (M=9.7 years, SD=1.0 years; *t*(113)=-1.92, *p*=0.57).

Moreover, broadly the same between-group differences between term-born and VP children were observed in the full sample and the sub-sample included in the DDM analysis. Compared with term-born children VP children were significantly older and had significantly lower IQ but were well-matched on other variables (see Table SA1). However, in contrast to the full sample, in the DDM analysis sub-sample VP children had significantly more severe parent-rated inattention, as measured by the SWAN, than those in the term-born sample.

*Table SA1:* *Sample characteristics for children included and excluded in the DDM analysis*

|  | **Full sample** | | | **DDM sub-sample** | | |
| --- | --- | --- | --- | --- | --- | --- |
|  | Very Preterm  (*n*=65^a^) | Term  (*n*=48^a^) | *p* | Very Preterm  (*n*=33^a^) | Term  (*n*=32^a^) | *p* |
| Participant characteristics | | | |  |  |  |
| Gestation (weeks) |  | | |  | | |
| Mean (*SD*) | 29^+6^ (1^+6^) | 40^+0^ (1^+1^) | - | 29^+4^ (1^+6^) | 40^+0^ (1^+1^) | - |
| Range | 26 to 32 | 37 to 42 |  | 26 to 32 | 37 to 42 |  |
| Age (years) |  | | |  | | |
| Mean (*SD*) | 10.1 (0.9) | 9.6 (1.0) | 0.006* | 9.6 (0.9) | 9.1 (1.1) | 0.031* |
| Range | 8.0 to 11.0 | 8.0 to 11.7 |  | 8.0 to 11.0 | 8.0 to 11.0 |  |
| FSIQ-2^b^ |  | | |  | | |
| Mean (*SD*) | 101.1 (13.9) | 111.1 (9.9) | <0.001* | 99.2 (14.0) | 111.6 (9.7) | <0.001* |
| Range | 67 to 131 | 83 to 127 |  | 67 to 122 | 89 to 127 |  |
| Score <70 *n*(%) | 1 (1.5%) | 0 |  | 1 (3.0%) | 0 |  |
| Demographics, *n*(%) | | | | | | |
| Female sex | 29 (44.6%) | 22 (45.8%) | 0.898 *n.s.* | 15 (45.5%) | 13 (46.4%) | 0.694 *n.s.* |
| Ethnicity |  | | |  | | |
| White | 47 (82.3%) | 42 (87.5%) | 0.855 *n.s.* | 23 (76.7%) | 27 (87.1%) | 0.283 *n.s.* |
| Mixed | 7 (12.3%) | 4 (8.3%) |  | 5 (16.7%) | 3 (9.7%) |  |
| Asian | 1 (1.8%) | 1 (2.1%) |  | 2 (6.7%) | 0 |  |
| Black | 1 (1.8%) | 1 (2.1%) |  | 0 | 1 (3.2%) |  |
| Chinese | 0 | 0 |  | 0 | 0 |  |
| Other | 1 (1.8%) | 0 |  | 0 | 0 |  |
| Socio-economic Status (SES) |  | | |  | | |
| Low SES | 12 (18.5%) | 13 (27.1%) | 0.074 *n.s.* | 7 (21.2%) | 10 (32.3%) | 0.379 *n.s.* |
| Middle SES | 25 (38.5%) | 9 (18.8%) |  | 11 (33.3%) | 6 (19.4%) |  |
| High SES | 28 (43.1%) | 26 (54.2%) |  | 15 (45.5%) | 15 (48.4%) |  |
| Conner’s 3 ADHD symptom scores | | | | | | |
| Conner’s 3 *T*-scores, mean *(SD)* |  | | |  | | |
| DSM ADHD/I | 62.11 (15.48) | 57.79 (13.51) | 0.136 *n.s.* | 62.00 (15.11) | 55.66 (12.66) | 0.072 *n.s.* |
| DSM ADHD/C | 61.63 (14.42) | 58.48 (14.08) | 0.399 *n.s.* | 61.94 (17.44) | 58.00 (15.11) | 0.335 *n.s.* |
| Inattention | 60.71 (15.64) | 57.13 (12.29) | 0.215 *n.s.* | 60.18 (15.26) | 55.53 (14.06) | 0.206 *n.s.* |
| Hyperactivity/ Impulsivity | 62.15 (16.24) | 59.06 (14.47) | 0.297 *n.s.* | 61.52 (17.43) | 59.03 (15.51) | 0.546 *n.s.* |
| IA-HI correlation, *r* | 0.78 | 0.83 | 0.233 *n.s.* | 0.75 | 0.86 | 0.109 *n.s.* |
| Conner’s 3 scores above clinical cut offs, *n*(%) |  | | |  | | |
| DSM ADHD/I | 22 (34.4%) | 12 (25.0%) | 0.286 *n.s.* | 12 (36.4%) | 7 (21.9%) | 0.199 *n.s.* |
| DSM ADHD/C | 21 (32.3%) | 13 (27.1%) | 0.549 *n.s.* | 10 (30.3%) | 10 (31.3%) | 0.934 *n.s.* |
| Inattention | 22 (33.8%) | 10 (20.8%) | 0.129 *n.s.* | 12 (36.4%) | 6 (18.8%) | 0.113 *n.s.* |
| Hyperactivity/ Impulsivity | 22 (33.8%) | 15 (31.3%) | 0.771 *n.s.* | 11 (33.3%) | 12 (37.5%) | 0.725 *n.s.* |
| SWAN symptom scores ^c^ | | | | | | |
| Inattention |  | | |  | | |
| Mean (*SD*) | -.068 (10.89) | -4.67 (12.22) | 0.080 *n.s.* | -0.70 (9.89) | -6.58 (12.23) | 0.038* |
| Range | -26 to 26 | -27 to 20 |  | -22 to 21 | -27 to 18 |  |
| Hyperactivity/ Impulsivity |  | | |  | | |
| Mean (*SD*) | -2.86 (11.13) | -6.71 (12.55) | 0.099 *n.s.* | -3.26 (8.69) | -8.35 (12.73) | 0.096 *n.s.* |
| Range | -27 to 25 | -27 to 27 |  | -21 to 14 | -27 to 27 |  |

Note: Age reflects chronological age for VP children. Continuous variables were compared using independent samples t-tests, rank variables were compared using Pearson's chi-square, correlations were compared using Fischer’s r-to-z. SD=standard deviation, FSIQ-2= two-subtest full scale intelligence quotient calculated using the Wechsler Abbreviated Scale for Intelligence. IA-HI correlation = correlation between inattentive (IA) and hyperactive-impulsive (HI) symptoms as measured using the Conner’s 3 subscale *T*-scores. **p*<0.05, *n.s.*= not significant. ^a^ accurate unless otherwise indicated. ^b^ Full sample: VP = 65, Term = 47. DDM sub-sample: VP = 33, Term = 30. ^c^ Full sample: VP = 57, Term = 48. DDM sub-sample: VP = 33, Term = 32.

*Figure SA1: Plot of RT quantiles (0.1, 0.3, 0.5, 0.7 and 0.9) for correct ‘go’ responses for observed and simulated data in very preterm and term children.*

*Figure SA2: Plot of RT and accuracy quantiles (0.1, 0.3, 0.5, 0.7 and 0.9) for correct ‘go’ responses for term and very preterm groups.*

*Table SA2:* *Partial correlation matrix between all inattention, task-performance measures and DDM parameters, controlling for age, with both groups combined*

|  | IA | CE | HR | RT | SDRT | *v* | *a* | *Ter* |
| --- | --- | --- | --- | --- | --- | --- | --- | --- |
| Inattention (IA) |  |  |  |  |  |  |  |  |
| Commission errors (CE) | 0.24  (0.057) |  |  |  |  |  |  |  |
| Hit rate (HR) | **-0.35**  (0.005) | **-0.56***  (<0.001) |  |  |  |  |  |  |
| Response time (RT) | 0.15  (0.235) | -0.09  (0.491) | -0.19  (0.126) |  |  |  |  |  |
| Response time variability (SD RT) | **0.32**  (0.011) | 0.10  (0.417) | **-0.30** (0.017) | **0.61***  (<0.001) |  |  |  |  |
| Drift rate (*v*) | **-0.37**  (0.003) | **-0.48***  (<0.001) | **0.92***  (<0.001) | **-0.36**  (0.003) | **-0.58***  (<0.001) |  |  |  |
| Boundary (*a)* | 0.02  (0.873) | **-0.29**  (0.021) | **0.44***  (<0.001) | **0.37**  (0.003) | **0.64***  (<0.001) | 0.16  (0.202) |  |  |
| Non-decision time (*Ter*) | -0.11  (0.405) | -0.12  (0.347) | -0.08 (0.553) | **0.53***  (<0.001) | **-0.33**  (0.007) | 0.04  (0.736) | **-0.40***  (<0.001) |  |

Note: *N*=65, except for correlations with inattention, where *n*=64. Values in bold meet alpha of *p*<0.05; Values with an asterisk meet Bonferroni-adjusted alpha of *p*<0.0017.

*Table SA3:* *Partial correlation matrix between all inattention, task-performance measures and DDM parameters, controlling for age, for each group separately.*

|  |  | Term-born (*n* = 32) | | | | | | | |
| --- | --- | --- | --- | --- | --- | --- | --- | --- | --- |
|  |  | IA | CE | HR | RT | SDRT | *v* | *a* | *Ter* |
| Very Preterm (*n* = 33) | Inattention (IA) |  | 0.32  (0.085) | **-0.42** (0.021) | 0.21  (0.265) | **0.47**  (0.008) | **-0.44**  (0.016) | 0.14  (0.473) | -0.16  (0.402) |
|  | Commission errors (CE) | 0.17  (0.362) |  | **-0.72*** (<0.001) | -0.13  (0.480) | 0.05  (0.771) | **-0.64***  (<0.001) | **-0.45**  (0.012) | -0.13  (0.483) |
|  | Hit rate (HR) | **-0.37**  (0.038) | **-0.35**  (0.047) |  | -0.20  (0.283) | -0.33  (0.069) | **0.94***  (<0.001) | **0.51**  (0.004) | -0.14  (0.445) |
|  | Response time (RT) | 0.13  (0.491) | -0.03  (0.891) | -0.19 (0.301) |  | **0.79***  **(<0.001)** | **-0.38**  (0.033) | **0.43**  (0.015) | **0.69***  (<0.001) |
|  | Response time variability (SD RT) | 0.16  (0.373) | 0.14  (0.442) | -0.22 (0.225) | **0.48**  (0.005) |  | **-0.55***  (<0.001) | **0.54**  (0.002) | 0.12  (0.515) |
|  | Drift rate (*v*) | **-0.36**  (0.041) | -0.30  (0.092) | **0.87***  (<0.001) | **-0.38**  (0.030) | **-0.59**  (<0.001) |  | 0.30  (0.096) | -0.17  (0.374) |
|  | Boundary (*a)* | -0.13  (0.484) | -0.15  (0.421) | **0.46**  (0.009) | 0.33  (0.067) | **0.71***  (<0.001) | 0.07  (0.690) |  | -0.13  (0.480) |
|  | Non-decision time (*Ter*) | 0.00  (0.984) | -0.11  (0.555) | -0.10 (0.605) | **0.39**  (0.027) | **-0.60***  (<0.001) | 0.15  (0.427) | **-0.57***  **(0.001)** |  |

Note: For correlations with inattention in the term-born children *n*=31. Values in bold meet alpha of *p*<0.05; Values with an asterisk meet Bonferroni-adjusted alpha of *p*<0.0017.

Table SA4 presents the results of a hierarchical stepwise regression analysis that included only standard task performance metrics. Age and group alone did not explain significant variance in parent-rated inattention (*F*(2,61)=2.522, *p*=0.089). The model was significantly improved with the addition of task-performance measures (Δ*R*^2^ =0.148, *p*=0.001), though only hit rate contributed enough un ue variance to be entered into Model 2, which explained 22.5% of the variance in inattention (Model 2; *F*(3,60)=5.793, *p*=0.002). Notably, with the inclusion of task-performance measures, group also explained significant unique variance in this model. Addition of group interaction terms in a third step using the forced entry technique did not significantly improve the model (Δ*R*^2^ = 0.002, *p*=0.915), and none of the group interaction terms explained sufficient unique variance to be entered at the third step using a stepwise entry technique, thus Model 2 was accepted as the final model.

*Table SA4:* Regression model for cognitive predictors of parent-rated inattention with only standard metrics and no DDM parameters

|  | Inattention | |
| --- | --- | --- |
|  | Model 1  *R*^2^=.076 | Model 2  *R*^2^=.225**  Δ*R*^2^= .148*** |
| Predictor | *β* | *β* |
| Group | .230 | .295* |
| Age | .099 | .110 |
| Hit rate |  | -0.391*** |
| RT variability |  | - |

Note: **p<*0.05; ** *p*<0.01;*** *p*<0.001. - = did not meet criteria for stepwise entry model selection.
